# Ni_3_V_2_O_8_ Marigold Structures with rGO Coating for Enhanced Supercapacitor Performance

**DOI:** 10.3390/mi15070930

**Published:** 2024-07-20

**Authors:** Manesh A. Yewale, Pritam J. Morankar, Vineet Kumar, Aviraj M. Teli., Sonali A. Beknalkar, Suprimkumar D. Dhas, Dong-Kil Shin

**Affiliations:** 1 School of Mechanical Engineering, Yeungnam University, Gyeongsan 38541, Republic of Korea; maneshphd@gmail.com (M.A.Y.);; 2 School of Chemical Engineering, Yeungnam University, 280 Daehak-ro, Gyeongsan 38541, Republic of Korea; 3 Division of Electronics and Electrical Engineering, Dongguk University—Seoul, 30 Pildong-ro, Jung-gu, Seoul 04620, Republic of Korea; 4 Department of Electronic Engineering, Institute for Wearable Convergence Electronics, Kyung Hee University, 1732 Deogyeong-daero, Giheung-gu, Yongin 17104, Republic of Korea

**Keywords:** Ni_3_V_2_O_8_, Ni_3_V_2_O_8_-rGO nanoparticles, hydrothermal synthesis, FESEM, TEM, XPS, supercapacitor

## Abstract

In this work, Ni_3_V_2_O_8_ (NVO) and Ni_3_V_2_O_8_-reduced graphene oxide (NVO-rGO) are synthesized hydrothermally, and their extensive structural, morphological, and electrochemical characterizations follow subsequently. The synthetic materials’ crystalline structure was confirmed by X-ray diffraction (XRD), and its unique marigold-like morphology was observed by field emission scanning electron microscopy (FESEM). The chemical states of the elements were investigated via X-ray photoelectron spectroscopy (XPS). Electrochemical impedance spectroscopy (EIS), Galvanostatic charge–discharge (GCD), and cyclic voltammetry (CV) were used to assess the electrochemical performance. A specific capacitance of 132 F/g, an energy density of 5.04 Wh/kg, and a power density of 187 W/kg were demonstrated by Ni_3_V_2_O_8_-rGO. Key electrochemical characteristics were b = 0.67; a transfer coefficient of 0.52; a standard rate constant of 6.07 × 10^−5^ cm/S; a diffusion coefficient of 5.27 × 10^−8^ cm^2^/S; and a series resistance of 1.65 Ω. By employing Ni_3_V_2_O_8_-rGO and activated carbon, an asymmetric supercapacitor with a specific capacitance of 7.85 F/g, an energy density of 3.52 Wh/kg, and a power density of 225 W/kg was achieved. The series resistance increased from 4.27 Ω to 6.63 Ω during cyclic stability tests, which showed 99% columbic efficiency and 87% energy retention. The potential of Ni_3_V_2_O_8_-rGO as a high-performance electrode material for supercapacitors is highlighted by these findings.

## 1. Introduction

As the energy crisis continues to worsen, energy storage that makes use of renewable energy sources has emerged as one of the most promising strategies for mitigating the effects of both the energy crisis and environmental degradation. There are a variety of energy storage devices that are currently available, including lithium-ion batteries, sodium-ion batteries, zinc-ion batteries, and others. Supercapacitors have garnered more attention than the other types of capacitors due to the distinctive qualities that they possess [1,2]. Supercapacitors are highly beneficial for prolonging battery life and are used in a wide range of applications, such as heavy machinery, hybrid autos, and the development of small-scale electronic equipment [3,4]. Supercapacitors possess numerous benefits, such as a prolonged lifespan and a high power density, in addition to their uncomplicated charge storage theory and construction. Supercapacitors are additionally cost effective, secure, and require minimal maintenance [5]. Supercapacitors are commonly made using transition metal oxides, carbon, and conducting electrical polymers as active electrode materials [6,7,8]. Electric double-layer capacitors (EDLCs) and pseudocapacitors (PCs) are the two types of supercapacitor electrode materials. The PC mechanism is demonstrated by metal oxides and metal chalcogenides, whereas the EDLC mechanism is represented by carbon-based materials such as rGO and CNT. Each of these materials possesses distinct electrical, chemical, and structural properties that impact the lifespan and ultimate performance of the supercapacitor [9]. Among them vanadates, which exhibit robust pseudocapacitive activity, have been used as electrode materials [10]. Vanadium-based materials exhibit exceptional chemical stability and a broad range of oxidation states, resulting in excellent electrochemical performance in supercapacitors [11]. Vanadium oxide is a highly promising electroactive redox material for use in supercapacitors due to its abundant availability, diverse oxidation states, and cost effectiveness [12]. Nickel oxides possess exceptional attributes such as a high theoretical specific capacity of 1292 C/g, low toxicity, high capacitance, low production cost, an ecologically friendly nature, and a charge storage mechanism known as pseudocapacitance [13,14]. The nickel and vanadium oxides possess unique characteristics. Therefore, nickel vanadium oxides are considered highly promising materials for usage in supercapacitors due to their wide range of oxidation states, cheap production cost, and ease of synthesis [15,16,17,18]. Nickel vanadium oxide nanoparticles are synthesized using a different synthesis methods such as the solvothermal/hydrothermal method, the sonochemical method, co-precipitation, and sol–gel, etc. [19,20]. The nickel vanadium oxide (NVO) electrode’s electrical conductivity and surface area can be greatly increased by incorporating reduced grapheme oxide (rGO). In the present report, we opted for the hydrothermal method for the typical synthesis of NVO and NVO-rGO nanoparticles. Micro-structural and morphological analysis, elemental mapping analysis, and electrochemical performance analysis are studied, and it is concluded that synthesized NVO and NVO-rGO are promising candidates for supercapacitor application.

## 2. Experimental Section

Ni_3_V_2_O_8_ (NVO) and Ni_3_V_2_O_8_-rGO (NVO-rGO) composite nanoparticles were synthesized via a straightforward hydrothermal process employing ammonium fluoride (AF) and urea (U). To synthesize the Ni_3_V_2_O_8_ nanoparticles, a solution was prepared by dissolving 0.1 M of nickel nitrate and 0.05 M of vanadium chloride in 40 mL of solvent in a 250 mL beaker. The solution was stirred regularly for 30 min to ensure homogeneity. Subsequently, a solution containing 1.2 M urea and 0.6 M ammonium fluoride was added to the previous solution and stirred for an additional 30 min to achieve homogeneity. The entire solution was thereafter transferred into a hydrothermal stainless steel reactor with a capacity of 100 mL. The reactor was carefully sealed and then placed in an oven set at a temperature of 140 °C for a reaction period of 12 h. Following the completion of the reaction, the reactor underwent natural cooling, and the resulting product was filtered using filter paper. The product had many washes with ethanol and water, each performed more than four times. Subsequently, it was dried in an oven at a temperature of 60 °C for the duration of one night. Finally, the product was annealed at a temperature of 400 °C for a period of 4 h. The product that underwent the process of annealing was given the name NVO-AFU. The synthesis of the Ni_3_V_2_O_8_-rGO product followed a similar procedure, except for the inclusion of 40 mg of graphene oxide (GO) at the beginning. The name given to this product is NVO-AFU-rGO. Ultimately, the products were utilized for additional investigations into their structural, surface, elemental, and electrochemical capabilities. These investigations were conducted on both the powdered form of the goods and the electrodes prepared from them, using the same naming convention.

PANalytical XRD with CuKα radiation was used to investigate the crystal structure and phase development of the microparticles. Field-emission scanning electron microscopy (FE-SEM; S-4800 HITACHI, Ltd., Chiyoda, Japan) examined nanoparticle surface morphology and elemental mapping. Surface chemical composition was investigated by XPS (K-alpha, Thermo Scientific, Altrincham, UK). A ZIVE SP5 electrochemical workstation was used to perform electrochemical measurements, including CV, EIS, GCD, LSV, and cyclic stability measurements, using a platinum counter electrode, microparticles as the working electrode, and an Ag/AgCl reference electrode (WonAtech, Seoul, Korea). To investigate the electrochemical performance of NVO-AFU and NVO-AFU-rGO in a three-electrode setup, NVO-AFU and NVO-AFU-rGO powders were used. The Ni foam was ultrasonically cleaned for 30 min with acetone, ethanol, and water. Slurries were prepared utilizing active material (NVO-AFU and NVO-AFU-rGO powders), PVDF, and carbon black in an 80:10:10 ratio. The prepared slurries were drop-coated onto pre-cleaned Ni foam and dried at 60 °C overnight. For the two-electrode configuration, NVO-AFU-rGO electrodes, termed the positive electrodes, were prepared in the same way, while activated carbon electrodes were prepared in the same way but with activated carbon instead of NVO-AFU-rGO powder. Activated carbon served as the negative electrode in the two-electrode setup. To investigate the ASC device’s electrochemical performance, the positive and negative electrodes were wrapped in paraffin paper and separated with filter paper in a 2 M KOH electrolyte.

## 3. Result and Discussion

### 3.1. X-ray Diffraction Analysis

**Figure 1:**
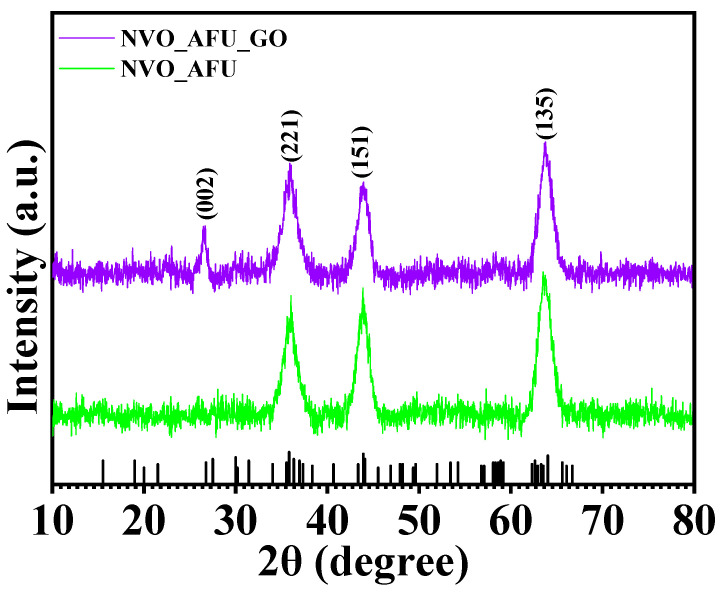
XRD spectra of Ni_3_V_2_O_8_ and Ni_3_V_2_O_8_-rGO nanoparticles.

X-ray diffraction (XRD) technology was used to assess the structural characteristics of nanomaterials, particularly the phase, purity, and crystallinity. Moreover, it serves as a potent analytical tool with the ability to provide precise information regarding the chemical structure of a given sample as well as the size of its unit cells. Figure 1 displays the X-ray diffraction pattern of the NVO_AFU and NVO_AFU_rGO samples, represented by the purple and blue colors, respectively. The presence of diffraction planes at 35.7°, 43.8°, and 63.4°, corresponding to the crystallographic indices (221), (151), and (135), respectively, indicates the synthesis of the NVO_AFU phase. The presence of an additional diffraction plane (002) at an angle of 26.3° in the NVO_AFU_rGO sample indicates the successful development of the NVO_AFU_rGO composite. The diffraction peak observed at an angle of 26.3° corresponds to the (002) plane, which is the distinctive peak of reduced graphene oxide (rGO), similar to that reported by V. Chellappa et al. [21]. The establishment of the NVO_AFU and NVO_AFU_rGO phases is confirmed by JCPDS card #01-070-1394. The NVO possesses an orthorhombic crystal structure with the space group Cmca (space group number: 64). The NVO crystal has lattice parameters of a = 5.93 Å, b = 11.42 Å, and c = 8.24 Å.

### 3.2. Field Electron Scanning Electron Microscopy (FESEM) Analysis

**Figure 2:**
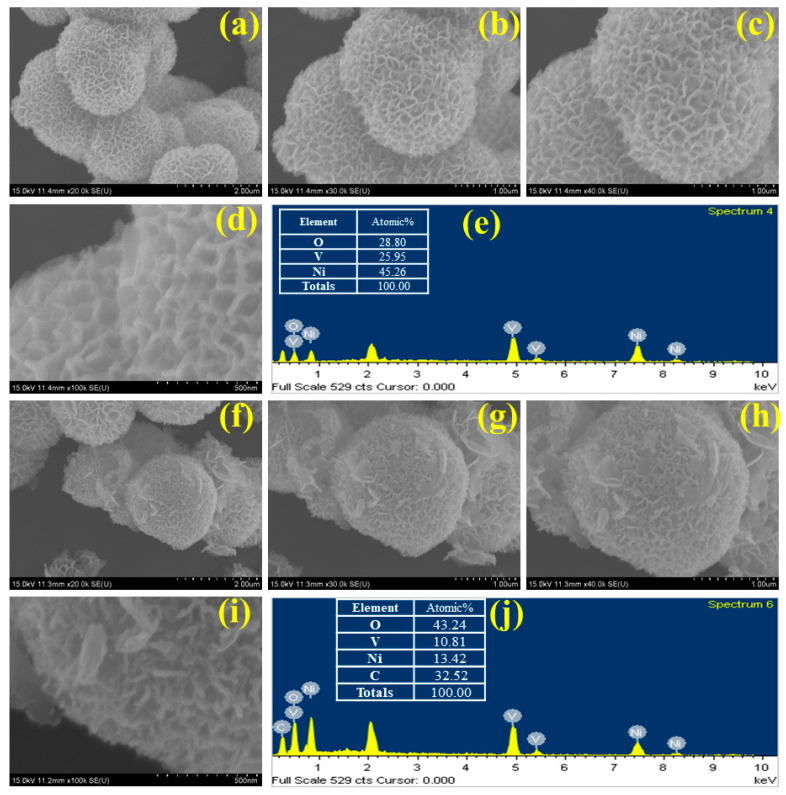
FESEM micrograph of Ni_3_V_2_O_8_ (**a**–**d**) and Ni_3_V_2_O_8_-rGO (**f**–**i**) nanoparticles, EDS spectra with elemental composition of Ni_3_V_2_O_8_ (**e**) and Ni_3_V_2_O_8_-rGO (**j**).

The surface morphology of the synthesized sample has been investigated using field electron scanning electron microscopy (SEM), while the elemental composition was analyzed using energy-dispersive X-ray spectroscopy (EDS). Figure 2a–d displays the surface characteristics of NVO_AFU nanoparticles at magnifications of ×20k, 30k, 40k, and 100k. Figure 2a–d clearly illustrate that the NVO_AFU nanoparticles possess a homogeneous marigold flower-like structure with an average diameter of approximately 2 μm. Figure 2f–i display the scanning electron microscope (SEM) images of the NVO_AFU_rGO composite at magnifications of 20k, 30k, 40k, and 100k. Figure 2f–i clearly show the visible presence of rGO nano-sheets on the pellet of NVO_AFU nanoparticles. This verifies the successful synthesis of the NVO_AFU_rGO composite. Some aggregation is observed in the SEM micrographs of both NVO_AFU and NVO_AFU_rGO nanoparticles. Figure 2e,j display the EDS spectra of the NVO_AFU and NVO_AFU_rGO composites, respectively, along with the elemental composition inset. Figure 2 provides information on the materials. This validates the formation of the stoichiometric phase in the NVO_AFU and NVO_AFU_rGO composites. The energy-dispersive X-ray spectroscopy (EDS) results in Figure 2e show that the stoichiometric ratio of Ni:V:O in NVO_AFU is 45.26:25.95:28.80. Figure 2j shows that the Ni:V:C:O ratio for NVO_AFU_rGO is 13.42:10.81:43.24:35.52 stoichiometrically. This elemental mapping result provides additional confirmation of the creation of the NVO_AFU and NVO_AFU_rGO composite phases, without any presence of impurities. These findings provide more evidence to validate the XRD analysis.

### 3.3. XPS Analysis

**Figure 3:**
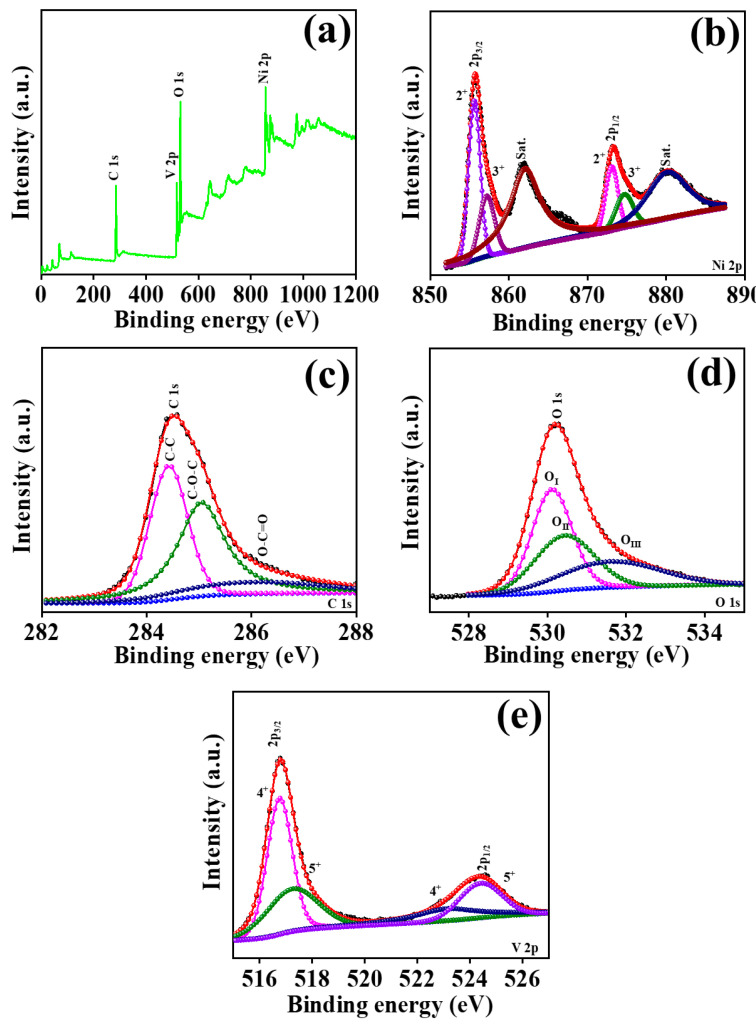
XPS spectra Ni_3_V_2_O_8_-rGO, survey scan (**a**), Ni 2p (**b**), C 1s (**c**), O 1s (**d**), V 2p (**e**).

The surface chemistry of the as-synthesized NVO_rGO nano-composite was investigated by X-ray photoelectron spectroscopy (XPS). The XPS approach was used in Figure 3 to find out the binding energies of different chemical states of elements found on the surface of the NVO_rGO sample. Figure 3a displays the XPS survey scan of the NVO_rGO sample, which was produced using hydrothermal methods. The scan covers the energy range of 0–1200 eV.

The peaks seen in the survey spectra at 285.90 eV, 529.13 eV, 854.18 eV, and 517.07 eV indicate the presence of carbon, oxygen, nickel, and vanadium in the NVO_rGO composite that was formed. The Gaussian and Lorentzian peak fitting analysis approach was employed to investigate the different oxidation states of nickel, vanadium, oxygen, and carbon. The XPS spectra for the Ni2p orbital are displayed in Figure 3b. The saturation peak is represented by two moderately intense peaks seen at binding energies of 861.65 eV and 880.25 eV. Two prominent peaks are detected at 855.61 eV and 872.34 eV, representing the Ni 2p_3/2_ and Ni 2p_1/2_ energy levels, respectively. The distinctive peaks of Ni^2+^ are assigned to energy levels of 855.59 eV and 873.02 eV for Ni 2p_3/2_ and Ni 2p_1/2_, respectively. The distinctive peaks of Ni^3+^ are ascribed to occur at 857.85 eV and 874.79 eV for the Ni 2p_3/2_ and Ni 2p_1/2_ oxidation states, respectively [22,23,24]. Figure 3c displays the spectra pertaining to the C 1s area. The peaks detected at binding energies of 284.04 eV, 285.05 eV, and 286.18 eV in the C 1s region correspond to the carbonate groups C-C, C-O-C, and O-C=O, respectively [25,26]. Figure 3d shows the measured peaks corresponding to the binding energies of 530.09 eV, 530.46 eV, and 531.76 eV for the oxygen atoms O_I_, O_II_, and O_III_, respectively, in the O 1s area of transition metal (oxy) hydroxides [27,28,29]. Figure 3e displays the V2p XPS spectra of the NVO_rGO nano-composite. The V 2p XPS spectra exhibit two clearly distinguishable peaks at 516.77 eV and 524.37 eV, which correspond to V 2p_3/2_ and V 2p_1/2_, respectively. The V 2p deconvoluted spectra exhibited four different peaks: 516.74 eV and 523.11 eV associated with the V^4+^ oxidation state and 517.49 eV and 524.43 eV related to the V^5+^ oxidation state [30,31,32,33,34,35]. The XPS results are consistent with the XRD and EDS results, providing confirmation of the presence and creation of Ni_3_V_2_O_8_-rGO.

### 3.4. Electrochemical Study

The distinctive morphology of Ni_3_V_2_O_8_ nanoparticles and Ni_3_V_2_O_8_-rGO composites, synthesized through the utilization of ammonium fluoride and urea, and an electrochemical study of the prepared electrode were investigated using a three-electrode setup for the purpose of energy storage in a 2 M KOH electrolyte. The data exhibit the CV, GCD, and EIS spectra of the Ni_3_V_2_O_8_ nanoparticles and Ni_3_V_2_O_8_-rGO composites.

**Figure 4:**
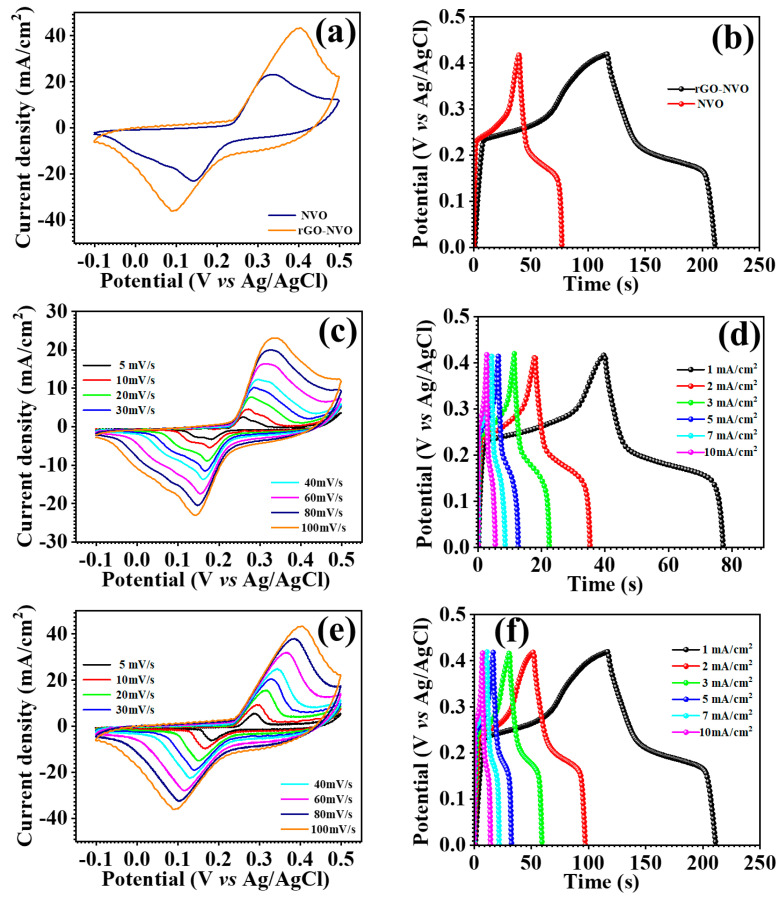
Comparative CV (**a**), GCD (**b**) profile, Ni_3_V_2_O_8_ and Ni_3_V_2_O_8_-rGO, CV (**c**,**e**) profile at different scan rate, GCD (**d**,**f**) at different current density of Ni_3_V_2_O_8_ and Ni_3_V_2_O_8_-rGO electrode.

The CV measurements were taken at a different scan rate of 5–100 mV/s, ranging from −0.1 to 0.5 V, with the Ag/AgCl electrode as the reference, as shown in Figure 4c,e. The Ni_3_V_2_O_8_ nanoparticles and Ni_3_V_2_O_8_-rGO composite electrodes both display two prominent redox peaks for oxidation and reduction, which indicate the characteristic electrochemical feature of a faradic redox process. The data illustrate the cyclic voltammetry (CV) characteristics of the Ni_3_V_2_O_8_ nanoparticles and Ni_3_V_2_O_8_-rGO composites as the scan rate increases from 5 to 100 mV/s. The two redox peaks are distinctly detected, with the anodic peak gradually shifting towards a positive direction and the cathodic peak shifting towards a negative direction, in addition to an increase in the peak current with the rise in scan rate. The Ni_3_V_2_O_8_-rGO composite electrode exhibits enhanced peak currents and a greater area under the CV profile, as shown in Figure 4a, suggesting faster faradic processes, reduced interfacial resistance, and increased specific capacitance in comparison to the Ni_3_V_2_O_8_ electrode. The enhancement is probably a result of the augmented conductivity of the electrode material facilitated by the rGO. The specific capacitances of the Ni_3_V_2_O_8_ nanoparticles and Ni_3_V_2_O_8_-rGO composite electrodes were calculated by analyzing the GCD profiles of the electrodes. Figure 4b illustrates the contrasting GCD patterns of the Ni_3_V_2_O_8_ nanoparticles and Ni_3_V_2_O_8_-rGO composites. The Ni_3_V_2_O_8_-rGO composite electrode demonstrates extended durations for both charging and discharging in comparison to the Ni_3_V_2_O_8_ electrode. The addition of rGO in the Ni_3_V_2_O_8_-rGO composite results in a greater specific capacitance compared to the Ni_3_V_2_O_8_ electrode. The electrodes’ specific capacitance (C_s_), specific energy density (ED_s_), and power density (PD_s_) were determined using Equations (1)–(3) [36,37,38]. The measured specific capacitance was determined to be 132 F/g, with an energy density of 5.04 Wh/kg and a power density of 187.14 W/kg at a current density of 1 mA/cm^2^. Figure 4d,f also display the GCD profiles of the Ni_3_V_2_O_8_ nanoparticles and Ni_3_V_2_O_8_-rGO composite electrodes under various current densities.

(1) $$\mathrm{Specific}\;\mathrm{Capacitance}\;(Cs)=\frac{I\times ∆t}{∆m\times ∆V}$$

(2) $$\mathrm{Energy}\;\mathrm{Density}\;(EDs)=\frac{C_{s}\times {∆V}^{2}}{7.2}$$

(3) $$\mathrm{Power}\;\mathrm{Density}\;(PDs)=\frac{{ED}_{s}\times 3600}{∆t}$$

where *I*, ∆t,∆m and∆V are the current density, discharging time, loading mass and potential window of the GCD profile.

The composite electrode benefits from the marigold-like surface microstructure and the incorporation of rGO belts, resulting in improved capacitive performance and an impressive electrochemical energy storage system. In addition, the electrochemical impedance spectroscopy (EIS) technique was used to investigate the reaction kinetics and conductivity of both the Ni_3_V_2_O_8_ nanoparticle and Ni_3_V_2_O_8_-rGO composite electrodes.

**Figure 5:**
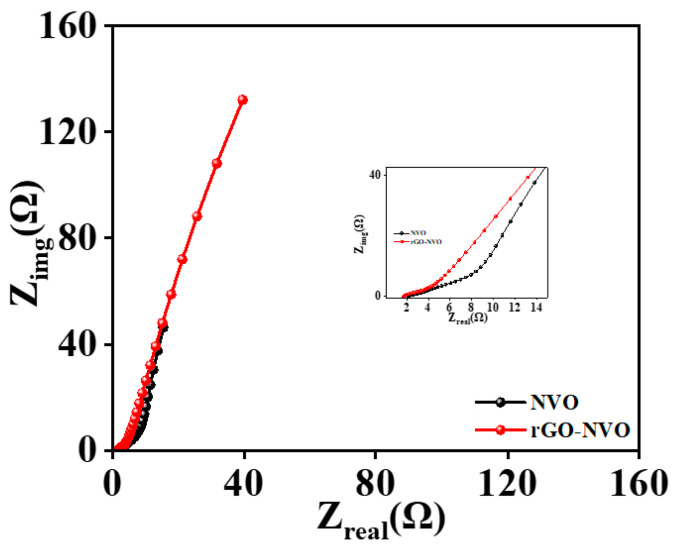
Electrochemical impedance spectra of Ni_3_V_2_O_8_ and Ni_3_V_2_O_8_-rGO electrode.

**Figure 6:**
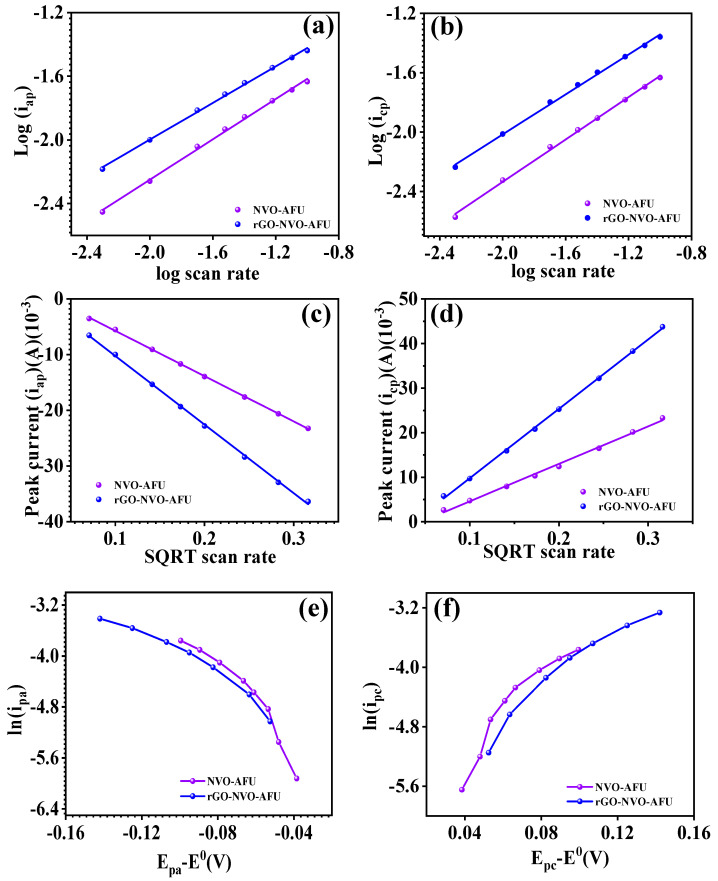
Log (*i_p_*) vs. Log (*v*) (**a**,**b**), *ip* vs. √v (**c**,**d**) and ln (*ip*) vs. E_pc_ *E*^0^ of Ni_3_V_2_O_8_ and Ni_3_V_2_O_8_-Rgo.

This analysis sought to assess the interaction between the electrode and electrolyte. The EIS spectra of the Ni_3_V_2_O_8_ nanoparticle and Ni_3_V_2_O_8_-rGO composite electrodes were measured in the frequency range of 1 MΩ to 0.1 Ω at an AC voltage of 5 mV, as shown in Figure 5. The region where the EIS spectrum intersects the x-axis represents the series resistance (R_s_) of the electrodes. The Ni_3_V_2_O_8_ and Ni_3_V_2_O_8_-rGO composite electrodes had series resistance values of 2.21 Ω and 1.65 Ω, respectively. The Ni_3_V_2_O_8_-rGO composite electrode exhibits superior electrochemical performance compared to the Ni_3_V_2_O_8_ electrode. This is likely attributed to the presence of rGO belts, which enhance conductivity by overlapping the marigold surface microstructure, as observed in the FESEM microstructure analysis. The linear trend observed in the EIS spectra signifies the ideal capacitive behavior and enhanced ion diffusion in the electrolyte. In addition, the kinetics of the electrochemical process were investigated by analyzing the b value, transfer coefficient (α), standard rate constant (*k*^0^), and diffusion coefficient (*D*) of the electrode material [39,40,41,42,43]. The CV profiles of the Ni_3_V_2_O_8_ and Ni_3_V_2_O_8_-rGO composite electrodes were utilized for the computation of these quantities. The b value of the electrodes was determined by plotting the logarithm of the current density (*i_p_*) against the logarithm of the scan rate (*v*), as shown in Figure 6a,b and Equation (4) [39,40]. The slope of this plot corresponds to the b value. The *b* value denotes the character of the present contribution, where a value of 0.5 signifies diffusion and a value of 1.0 signifies capacitive contributions.

(4) $$i={av}^{b}$$

(5) $$\frac{i}{\surd v}=2.69\times A\times C\times \surd D\times \surd n$$

(6) $$i_{p=0.227ACFnk^{0}exp\left[\frac{\propto nF}{RT}(E_{p}-E^{0})\right]}$$

where *b*, *A*, *C*, *n*, *D*, *F*, *R*, *T*, *E_p_* and *E*^0^ are the *b* value, area of the electrode, concentration of the electrolyte, number of electron present, diffusion coefficient, Faraday’s constant, universal gas constant, temperature, peak potential and formal potential.

The b value for the Ni_3_V_2_O_8_-rGO electrode was measured to be 0.71 and 0.63, but the Ni_3_V_2_O_8_-rGO composite exhibited values of 0.67 and 0.57 for the cathodic and anodic peaks, respectively. Both values fall within the range of 0.5 to 1.0, indicating a blend of capacitive and diffusion influences. The Ni_3_V_2_O_8_-rGO composite has a greater diffusion contribution, potentially attributed to the marigold surface microstructure with rGO bands that facilitate ion diffusion throughout the charging and discharging cycle. In supercapacitors, where the current distribution in charge storage either occurs through capacitive or diffusion contributions, ion diffusion is an essential concept for energy storage applications. Supercapacitors are used to store energy. Ions contribute to the diffusion process, which is represented by the diffusion coefficient. This process is what causes the diffusion contribution. The diffusion coefficients of the electrode material were estimated from the CV profile by plotting the peak current (*i_p_*) versus the square root of the scan rate (*v*). This results in the calculation of the diffusion coefficient. Both the cathodic and anodic peaks for both electrodes are depicted in Figure 6c,d as a plot of the peak current vs. the square root of the scan rate. Equation (5) is used to compute the diffusion coefficient, which results in the values of 2.28 × 10^−8^ cm^2^/S and 2.45 × 10^−8^ cm^2^/S for the Ni_3_V_2_O_8_ electrode and 5.27 × 10^−8^ cm^2^/S and 8.3.7 × 10^−8^ cm^2^/S for the Ni_3_V_2_O_8_-rGO composite for the anodic and cathodic peaks, respectively, during the computation [39,41]. It is likely that the marigold surface microstructure, which coincides with the wrapping of the rGO belt, contributes for the higher diffusion coefficient of the Ni_3_V_2_O_8_-rGO composite electrode. Through the utilization of the standard rate constant (k^0^) and the transfer coefficient (α), one may estimate the reaction that occurs between the electrode and the electrolyte. To determine the *k*^0^ value and the transfer coefficient, Figure 6e,f are used alongside with Equation (6) for the computation. Figure 6e,f illustrate the relationship between the ln(*i_p_*) and *E_p_*-*E*^0^ for both the cathodic and anodic values of both electrodes. Redox reactions, which can be reversible, irreversible, or quasi-reversible, are mechanisms that are responsible for charge storage. *k*^0^ is less than or equal to 10^−5^ for reversible redox reactions, *k*^0^ is more than or equal to 10^−5^ for irreversible redox reactions, and 10^−1^ is less than or equal to *k*^0^ for quasi-reversible redox processes [39,41,42,43,44]. The *k*^0^ values for the Ni_3_V_2_O_8_ electrode are 2 × 10^−5^ cm/S and 2.89 × 10^−5^ cm/S, and for the Ni_3_V_2_O_8_-rGO composite electrode, 6.07 × 10^−5^ cm/S and 4.82 × 10^−5^ cm/S for the anodic and cathodic peaks, respectively. Indicating that both electrodes display redox reactions through a mechanism that is quasi-reversible, these values are within the range of redox reactions that are considered to be quasi-reversible. Quasi-reversible redox processes are also represented by the transfer coefficient, which can range from 0.0 to 1.0 degrees. Both of the electrodes exhibit a quasi-reversible redox nature, as evidenced by the transfer coefficients of 0.76 and 0.52, respectively, for the Ni_3_V_2_O_8_ and Ni_3_V_2_O_8_-rGO composite forms of the material.

### 3.5. Asymmetric Supercapacitor (ASC)

The Ni_3_V_2_O_8_-rGO composite electrode demonstrates remarkable electrochemical performance in a three-electrode configuration study. To explore its practical application, the Ni_3_V_2_O_8_-rGO composite electrode was further investigated using a two-electrode configuration. An asymmetric supercapacitor (ASC) device was fabricated using the Ni_3_V_2_O_8_-rGO composite as the positive electrode and activated carbon (AC) as the negative electrode. The ASC assembly involved wrapping the Ni_3_V_2_O_8_-rGO composite electrode and the AC electrode together with paraffin paper, sandwiching filter paper between them, and using 2M KOH as the electrolyte. The electrochemical performance of the ASC was evaluated through cyclic voltammetry (CV) and galvanostatic charge–discharge (GCD) measurements at different scan rates, current densities, and potentials.

**Figure 7:**
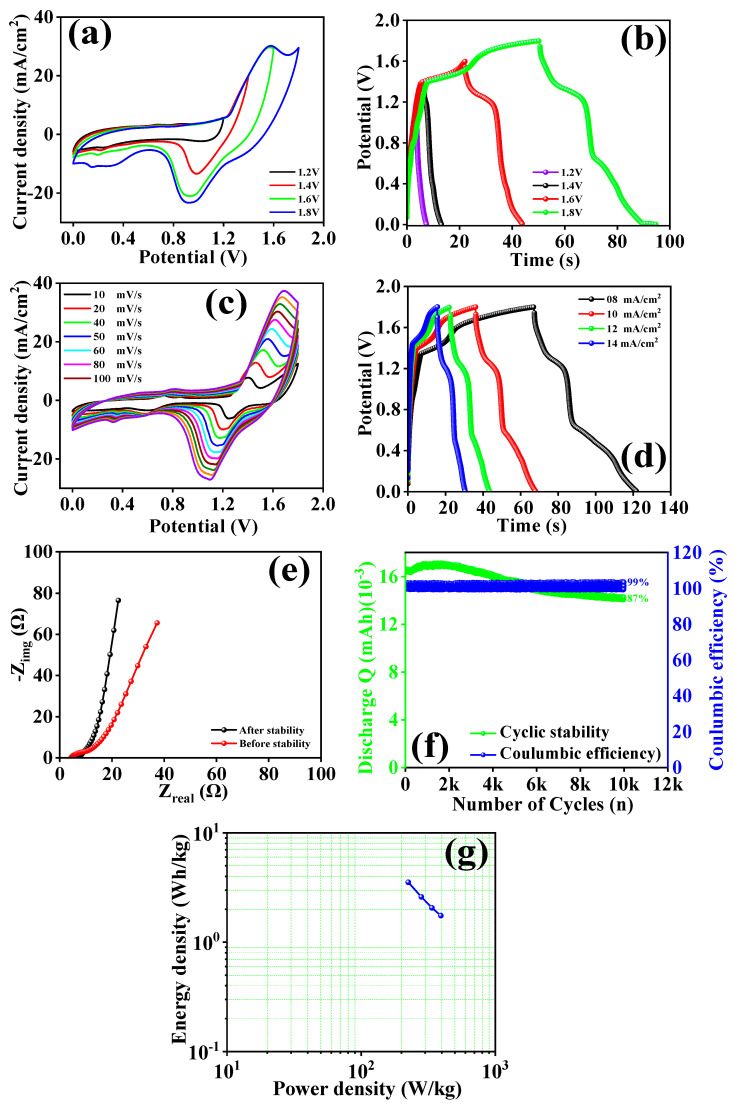
CV (**a**) profile at different potentials, GCD (**b**) profile at different potentials, CV (**c**) profile at different scan rates, GCD (**d**) profile at different current densities, EIS (**e**) spectra before and after stability, (**f**) stability and columbic efficiency over 10,000 GCD cycles, (**g**) Ragone plot of asymmetric supercapacitor.

Figure 7a,b illustrate the CV and GCD profiles of the ASC device across different potential ranges from 1.2 to 1.8 V. The CV profile remained stable without any noticeable changes, confirming that the optimal potential for the ASC is 1.8 V. Maintaining a potential of 1.8 V, the rate capability of the ASC was studied by performing CV measurements at various scan rates ranging from 10 to 100 mV/s, as shown in Figure 7c. The consistent increase in current with higher scan rates, without any alteration in the CV profile shape, demonstrated the strong capacitive performance of the device. Figure 7d shows GCD profiles of the ASC at a constant potential of 1.8 V with different current densities ranging from 8 to 14 mA/cm^2^. The absence of significant distortion with increasing current density indicates that the ASC can operate stably within the potential range of 1.2–1.8 V. Using Equations (1)–(3), the specific capacitance (C_s_), energy density (ED_s_), and power density (PD_s_) of the ASC were calculated [38,39,40]. The ASC exhibited a specific capacitance of 7.85 F/g, an energy density of 3.56 Wh/kg, and a power density of 225 W/kg at a current density of 8 mA/cm^2^. The specific capacitance of the ASC decreased with increasing current density due to the non-ideal diffusion nature, which limited the number of ions participating in the process over a given time while using less active material. Electrochemical impedance spectroscopy (EIS) was employed to analyze the electrode and electrolyte interaction of the ASC device before and after stability testing. Figure 7e shows the EIS spectra of the ASC before and after stability testing. In the high-frequency region, the intercept at the x-axis indicates the series resistance (R_s_). The ASC exhibited R_s_ values of 4.27 Ω before stability and 6.63 Ω after stability. The increase in R_s_ after stability might be attributed to surface modification following prolonged cycling. Additionally, EIS analysis provided insights into the charge transfer resistance (R_ct_) and the Warburg impedance, which related to ion diffusion. Before stability testing, the Nyquist plot of the ASC showed a smaller semicircle at high frequencies, indicating lower R_ct_, and a steeper slope in the low-frequency region, signifying efficient ion diffusion.

After 10,000 cycles, the semicircle’s diameter increased, and the slope in the low-frequency region decreased, suggesting that prolonged cycling slightly impeded the charge transfer and ion diffusion processes. The long-term practical capability of the ASC was assessed by performing GCD cyclic stability tests over 10,000 cycles. Figure 7f illustrates the cyclic stability of the ASC device. Initially, the capacitance of the ASC increased up to approximately 2000 cycles, likely due to the activation of the electrode material and the diffusion of ions within the electrode. Subsequently, the discharge capacity decreased with further cycling, possibly due to surface microstructure modification during the charging and discharging processes. After 7000 GCD cycles, only a minor decrease in discharge capacity was observed, resulting in approximately 87% cyclic stability over 10,000 cycles. The columbic efficiency of the ASC device was measured at 99% over 10,000 cycles in Figure 7f, indicating excellent cyclic stability and columbic efficiency, making the ASC a potential candidate for energy storage applications. The improved performance of the Ni_3_V_2_O_8_-rGO composite electrode in the ASC device can be attributed to the synergistic effects of the Ni_3_V_2_O_8_ nanoparticles and the rGO sheets. The rGO sheets enhance electrical conductivity and provide a robust framework for the Ni_3_V_2_O_8_ nanoparticles, facilitating faster electron transport and ion diffusion. The marigold-like surface microstructure of the Ni_3_V_2_O_8_ nanoparticles, combined with the high surface area and excellent conductivity of the rGO sheets, results in superior electrochemical performance. Furthermore, the ASC’s performance metrics, such as specific capacitance, energy density, and power density, are highly competitive with other state-of-the-art supercapacitor reported in the literature. The specific capacitance of 7.85 F/g, energy density of 3.56 Wh/kg, and power density of 225 W/kg underscore the potential of the Ni_3_V_2_O_8_-rGO composite electrode for high-performance energy storage devices. The outstanding cyclic stability and columbic efficiency of the ASC device, maintaining 87% capacitance retention and 99% efficiency over 10,000 cycles, highlight its robustness and reliability for long-term applications. The slight increase in series resistance and charge transfer resistance after prolonged cycling suggests that while the device remains highly efficient, further optimization of the electrode materials and device architecture could further enhance stability and performance. The Ni_3_V_2_O_8_-rGO composite electrode exhibits exceptional electrochemical properties in both three-electrode and two-electrode configurations. The successful integration of Ni_3_V_2_O_8_ nanoparticles with rGO sheets leads to enhanced capacitive performance, high energy and power densities, and excellent long-term stability. The development of ASCs using these composite electrodes holds significant promise for advanced energy storage systems, offering a viable solution for various applications requiring an efficient and durable supercapacitor. The comparative study were listed in Table 1.

## 4. Conclusions

The method of hydrothermal synthesis and comprehensive characterization of Ni_3_V_2_O_8_ and Ni_3_V_2_O_8_-reduced graphene oxide (Ni_3_V_2_O_8_-rGO) showed significant advances in the development of electrode materials for supercapacitors. An X-ray diffraction (XRD) investigation verified that the produced materials have a crystalline structure. Furthermore, field emission scanning electron microscopy (FESEM) shows a unique marigold-like shape, which suggests that the materials have a large surface area that is advantageous for electrochemical applications. The XPS examination yielded valuable information about the chemical states, thus enhancing our understanding of the material’s composition. The electrochemical investigations demonstrated the exceptional efficiency of Ni_3_V_2_O_8_-rGO, which displayed a specific capacitance of 132 F/g, an energy density of 5.04 Wh/kg, and a power density of 187 W/kg. The effective electrochemical kinetics of the system were highlighted by key metrics, including a b value of 0.67, a transfer coefficient of 0.52, a standard rate constant of 6.07 × 10^−5^ cm^2^/S, a diffusion coefficient of 5.27 × 10^−8^ cm/s, and a series resistance of 1.65 Ω. An asymmetric supercapacitor was constructed by combining Ni_3_V_2_O_8_-rGO and activated carbon (AC), resulting in a specific capacitance of 7.85 F/g, an energy density of 3.52 Wh/kg, and a power density of 225 W/kg. The device exhibited exceptional cyclic stability, with a columbic efficiency of 99% and 87% energy retention after 10,000 cycles. The series resistance increased moderately from 4.27 Ω to 6.63 Ω. The results confirm that Ni_3_V_2_O_8_-rGO has the capability to be used as an excellent electrode material, which can be applied in advanced energy storage systems of the future.

## Figures and Tables

**Table 1:** Comparative study of the ASC.

Sr. No.	Material	Energy Density (Wh/kg)	Power Density (W/kg)	Reference
1.	Ni_3_V_2_O_8_-rGO/AC	3.56	225	Present work
2.	NS-GF@RuO_2_//NS-GF@RuO_2_	2.93	1428	[45]
3.	MoO_2_@Cu@C	2.58	86	[46]
4.	Co_3_O_4_/graphene	2.4	300	[47]
5.	poly(Ani-co-Pip)/Vu@PSS	1.19	0.018	[48]

## Data Availability

All the relevant data are included in this published article.
